# A Systematic Review and Meta-Analysis of Artificial Intelligence Tools in Medicine and Healthcare: Applications, Considerations, Limitations, Motivation and Challenges

**DOI:** 10.3390/diagnostics14010109

**Published:** 2024-01-04

**Authors:** Hussain A. Younis, Taiseer Abdalla Elfadil Eisa, Maged Nasser, Thaeer Mueen Sahib, Ameen A. Noor, Osamah Mohammed Alyasiri, Sani Salisu, Israa M. Hayder, Hameed AbdulKareem Younis

**Affiliations:** 1 College of Education for Women, University of Basrah, Basrah 61004, Iraq; 2 Department of Information Systems-Girls Section, King Khalid University, Mahayil 62529, Saudi Arabia; teisa@kku.edu.sa; 3 Computer & Information Sciences Department, Universiti Teknologi PETRONAS, Seri Iskandar 32610, Malaysia; maged.nasser@utp.edu.my; 4 Kufa Technical Institute, Al-Furat Al-Awsat Technical University, Kufa 54001, Iraq; kin.thr@atu.edu.iq; 5 Computer Science Department, College of Education, University of Almustansirya, Baghdad 10045, Iraq; a.ameen63@uomustansiriyah.edu.iq; 6 Karbala Technical Institute, Al-Furat Al-Awsat Technical University, Karbala 56001, Iraq; osama.alyasiri@atu.edu.iq; 7 Department of Information Technology, Federal University Dutse, Dutse 720101, Nigeria; sani.salisu@fud.edu.ng; 8 Qurna Technique Institute, Southern Technical University, Basrah 61016, Iraq; israa.mh@stu.edu.iq; 9 Department of Cybersecurity, College of Computer Science and Information Technology, University of Basrah, Basrah 61016, Iraq; hameed.younis@uobasrah.edu.iq

**Keywords:** ChatGPT, cellular imaging, medicine, healthcare, image, dental, disease, radiology and sonar, pharmaceutical

## Abstract

Artificial intelligence (AI) has emerged as a transformative force in various sectors, including medicine and healthcare. Large language models like ChatGPT showcase AI’s potential by generating human-like text through prompts. ChatGPT’s adaptability holds promise for reshaping medical practices, improving patient care, and enhancing interactions among healthcare professionals, patients, and data. In pandemic management, ChatGPT rapidly disseminates vital information. It serves as a virtual assistant in surgical consultations, aids dental practices, simplifies medical education, and aids in disease diagnosis. A total of 82 papers were categorised into eight major areas, which are G1: treatment and medicine, G2: buildings and equipment, G3: parts of the human body and areas of the disease, G4: patients, G5: citizens, G6: cellular imaging, radiology, pulse and medical images, G7: doctors and nurses, and G8: tools, devices and administration. Balancing AI’s role with human judgment remains a challenge. A systematic literature review using the PRISMA approach explored AI’s transformative potential in healthcare, highlighting ChatGPT’s versatile applications, limitations, motivation, and challenges. In conclusion, ChatGPT’s diverse medical applications demonstrate its potential for innovation, serving as a valuable resource for students, academics, and researchers in healthcare. Additionally, this study serves as a guide, assisting students, academics, and researchers in the field of medicine and healthcare alike.

## 1. Introduction

Artificial intelligence (AI) has emerged as a powerful tool with transformative potential across various sectors, and the field of medicine and healthcare is no exception. One remarkable application of AI in this realm is the development of large language models, such as ChatGPT, which have gained significant attention for their ability to generate human-like text based on prompts. ChatGPT’s versatile capabilities hold promise for reshaping medical practices, enhancing patient care, and revolutionising the way healthcare professionals interact with both patients and data [1,2,3,4,5,6,7,8,9,10,11,12,13,14,15,16,17,18,19,20,21,22,23,24,25,26,27,28,29,30,31,32].

OpenAI launched in last November 2022 the Chat Generative Pre-trained Transformer (ChatGPT) and revolutionised the approach in artificial intelligence to human–model interaction used for fields [4]. It was used in all areas: healthcare and management [5,6,7,8,9], aiding cosmetic orthognathic surgery consultation [10], revolutionising dental practices [8,11,12], medical scientific articles and education fields [6,9,13,14,15,16,17,18,19,20,21,22,23], support in disease diagnosis [24], revolutionising radiology, sonar imaging and writing assessment [13,25,26,27,28,29,30,31,32,33], pharmaceutical research and treatment [11,24,34,35,36,37,38], and navigating limitations and ethical considerations [18,19,31,35,38,39,40,41]. Addressing the open issues in AI applications, such as ChatGPT, particularly for medical use, involves the tackling of model interpretability. This is carried out to make the AI’s decision making transparent, especially in complex medical scenarios. Equally critical is the combatting of data bias in order to prevent healthcare disparities. There is also a pressing need for mechanisms that allow for continuous learning, which enables ChatGPT to stay abreast of the latest medical research and guidelines. Moreover, the management of integrating ChatGPT with existing healthcare IT ecosystems is crucial to guarantee seamless operation. Lastly, it is essential to strictly adhere to ethical and legal standards, with a focus on maintaining trust and compliance in healthcare applications through patient confidentiality and informed consent.

The innovative aspects of ChatGPT’s deployment in medicine include a variety of unique applications, such as assisting in the identification of rare diseases and providing support for men’s health, demonstrating the model’s versatility. Advanced methodologies are at the heart of its training and effectiveness evaluation, which are tailored specifically for healthcare contexts to ensure relevance and dependability. The work is fundamentally interdisciplinary, as it merges AI with expert medical insights in order to effectively tackle significant healthcare challenges. The aim of this synergy is to translate into tangible benefits, which include enhanced patient outcomes and streamlined healthcare processes. This, in turn, marks a substantial real-world impact on the medical field. The areas of ChatGPT’s application in medical fields and healthcare are as follows.

### 1.1. Aiding Cosmetic Orthognathic Surgery Consultations

Within the realm of surgical procedures, such as cosmetic orthognathic surgery, ChatGPT can function as a virtual assistant, offering patients crucial preoperative information. Potential candidates who are considering cosmetic orthognathic surgery may be seeking information regarding the procedure, recovery, risks, and benefits. ChatGPT can offer standardised and accurate responses, which prepare patients for their consultations and help them make informed decisions [5,6,7,8,9,10,42,43].

### 1.2. Enhancing Medical Education

Medical education can benefit significantly from AI-driven tools like ChatGPT. Medical students and professionals can engage with ChatGPT to access quick references, clarify doubts, and explore complex medical concepts. Its ability to explain intricate medical terminology in a comprehensible manner aids in knowledge acquisition, fostering continuous learning and improving medical literacy [6,9,13,14,15,16,17,18,19,20,21,22,23].

### 1.3. Support in Disease Diagnosis

The potential of ChatGPT as a diagnostic tool holds promise in the early detection of diseases. ChatGPT can generate potential differential diagnoses by analysing patient-reported symptoms and medical history, thereby aiding healthcare providers in narrowing down diagnostic possibilities. However, caution must be exercised as diagnosis requires domain-specific expertise [24].

### 1.4. Cellular Imaging, Revolutionizing Radiology, and Sonar Imaging

Cellular imaging pertains to the utilisation of diverse techniques and technologies for visualising and studying cells at the microscopic level. Scientists and researchers are allowed to examine the structure, function, and behaviour of individual cells or cell populations. Cellular imaging techniques comprise light microscopy, fluorescence microscopy, confocal microscopy, electron microscopy, and various other advanced imaging methods. These techniques are widely used in various fields, such as biology, medicine, and biotechnology, for the purpose of better understanding cellular processes, cell interactions, and disease mechanisms.

The interpretation of medical images, such as radiology and sonar scans, requires precision and accuracy. The potential of ChatGPT lies in its ability to analyse and generate descriptions for medical images, which can enhance the workflow of radiologists. It has the ability to provide initial observations, which can highlight regions of interest and assist radiologists in their analyses [13,25,26,27,28,29,30,31,32,33].

### 1.5. Pharmaceutical Research and Treatment

In the realm of pharmaceutical research, ChatGPT can contribute by sifting through vast volumes of scientific literature, identifying potential drug candidates, and suggesting innovative research directions. It can assist in drug discovery, research proposal writing, and summarising complex medical research, expediting the research process [11,24,34,35,36,37,38].

### 1.6. Navigating Limitations and Ethical Considerations

**Figure 1:**
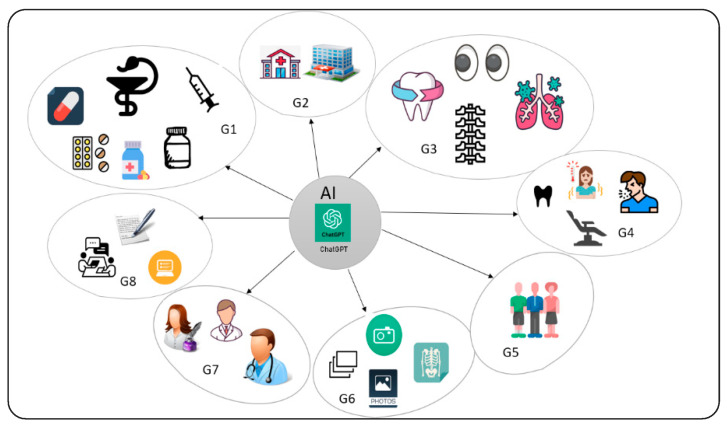
Categories and groups covered by all studies of the review. G1: treatment and medicine, G2: buildings and equipment, G3: parts of the human body and areas of the disease, G4: patients, G5: citizens, G6: cellular imaging, radiology, pulse, and medical images, G7: doctors and nurses, and G8: tools, devices and administration.

While ChatGPT offers numerous opportunities, it also poses certain limitations. The responses are based on the data on which it was trained, potentially resulting in biased or inaccurate information. Ethical concerns arise when content generated by AI is mistaken for the expertise of a human. The challenge of balancing the role of AI with the need for human judgement and expertise remains [18,19,27,31,35,38,39,40,41], as depicted in Figure 1.

Johnson, D et al. [5] utilised the accuracy and completeness of ChatGPT in answering medical queries by academic physician specialists. The results generally show accurate information with limitations, which suggests a need for further research and model development. Mohammad H et al. [13] conducted a hybrid panel discussion that focused on the integration of ChatGPT, a large language model, in the fields of education, research, and healthcare. The event gathered responses from attendees, both in-person and online, by utilising an audience interaction platform. According to the study, approximately 40% of the participants had utilised ChatGPT, with a higher number of trainees compared to faculty members having experimented with it. Those individuals who had utilised ChatGPT demonstrated a heightened level of interest in its potential application across a multitude of contexts. Uncertainty was observed regarding its use in education, with pros and cons being discussed for its integration in education, research, and healthcare. The perspectives varied according to role (trainee, faculty, and staff), highlighting the need for further discussion and exploration of the implications and optimal uses of ChatGPT in these sectors. The study emphasises the significance of taking a deliberate and measured approach to adoption in order to mitigate potential risks and challenges. Sallam et al. [44] assessed the advantages and disadvantages of ChatGPT in healthcare education. While it is beneficial for learning, it lacks emotional interaction and poses plagiarism risks.

In conclusion, the integration of ChatGPT into medicine and healthcare holds significant potential to transform various aspects of patient care, education, diagnosis, and research. As technology continues its advancement, it becomes crucial to responsibly harness the capabilities of ChatGPT, while also addressing its limitations and ethical considerations. The exploration of ChatGPT’s applications across diverse medical domains emphasises its role as a catalyst for innovation and improvement in the medical landscape.

The main objectives of the study can be succinctly summarised as follows:

- To investigate the various applications of ChatGPT in healthcare, such as pandemic management, surgical consultations, dental practices, medical education, disease diagnosis, cellular imaging, sonar imaging, radiology, and pharmaceutical research.
- To investigate the potential benefits and risks of integrating ChatGPT in healthcare, assessing its impact on patient care, medical processes, and ethical considerations.
- To investigate the ethical issues and the need to balance AI’s role in healthcare settings with human judgement.
- To offer perspectives and recommendations for the responsible adoption and use of ChatGPT in medicine in general, and cellular imaging in particular, while addressing limitations and ethical concerns.

## 2. Materials and Methods

**Figure 2:**
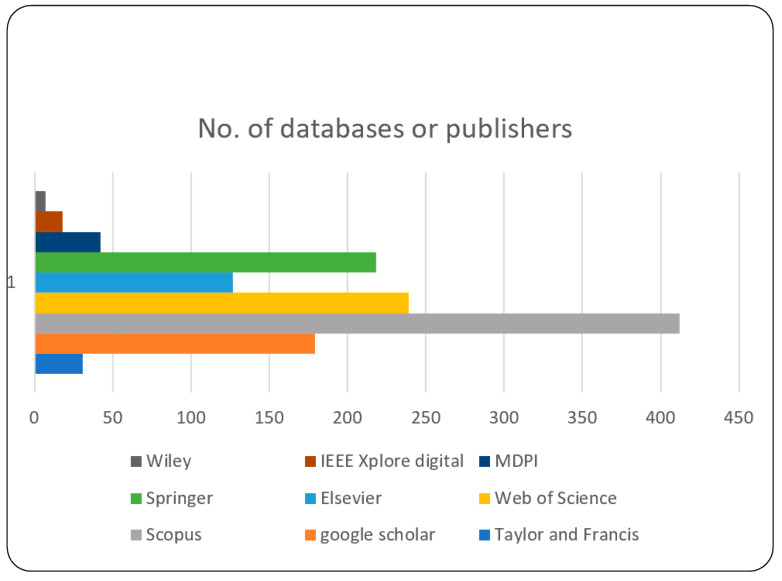
No. of databases/publishers.

The field of this study is covered by very important query words (keywords), namely, ChatGPT with “Medicine”, “Healthcare”, “Tackles”, “Pandemics/Infectious”, “A Cosmetic orthognathic surgery Consultation”, “dental Medical/Education”, “Disease, Radiology”, “sonar”, “pharmaceutical”, “Treatment”, “Diagnosis” and “patient care”, “Medical school”, “Ophthalmology”, “Digital Health”, and “operating room”. Our study is closed to English-language studies only. The following digital databases and publishers were selected to search for target papers, and the numbers of articles included the following form: Taylor and Francis (31) article, Google Scholar (179) article, Scopus (412) article, Web of Science (239) article, Elsevier (127) article, Springer (218) article, MDPI (41) article, IEEE Xplore digital (18) article, and Wiley (7) article, as shown in Figure 2.

The studies were chosen by conducting literature searches, which were then followed by three rounds of screening and filtering. In the initial iteration, using Mendeley software (1.19.4-win32), only publications that were published in the last eight months were gathered after eliminating duplicate articles.

**Figure 3:**
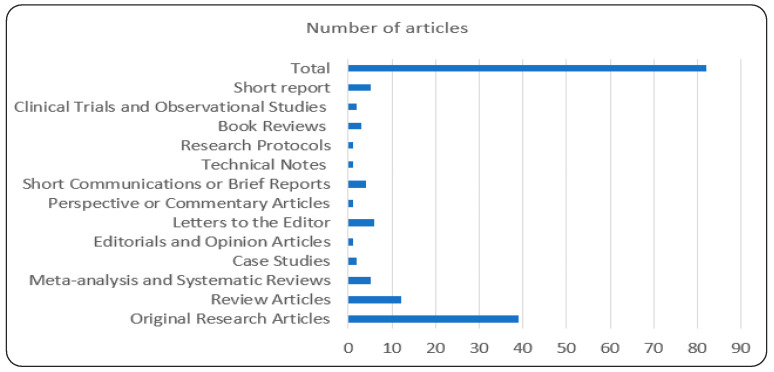
Distribution by type of articles to number of articles.

As part of this investigation, a systematic literature review was conducted using the PRISMA methodology (Supplementary Materials) [40,45,46,47,48,49,50]. We included all types of articles: original research articles, review articles, meta-analysis and systematic reviews, case studies, editorials and opinion articles, letters to the editor, perspective or commentary articles, short communications or brief reports, technical notes, research protocols, book reviews, clinical trials and observational studies, and short reports; the numbers included the following form (39, 12, 5, 2, 1, 6, 1, 4, 1, 1, 3, 2, and 5), respectively, as shown in Figure 3.

### 2.1. Inclusion Criteria

- The article should be original or reviewed, written in English, and published in English journals, conferences, case studies, editorials, opinion articles, and letters to the editor.
- The study specifies that it contains the specified keywords.
- Any relevant articles are published or in press between November 2022 and August 2023.
- The main point is on Chat GPT with specific words in the field of healthcare and health.
- The study includes the study’s objectives, design, applications, benefits, risks, concerns, limitations, study field/area, conclusions, and recommended actions.

**Figure 4:**
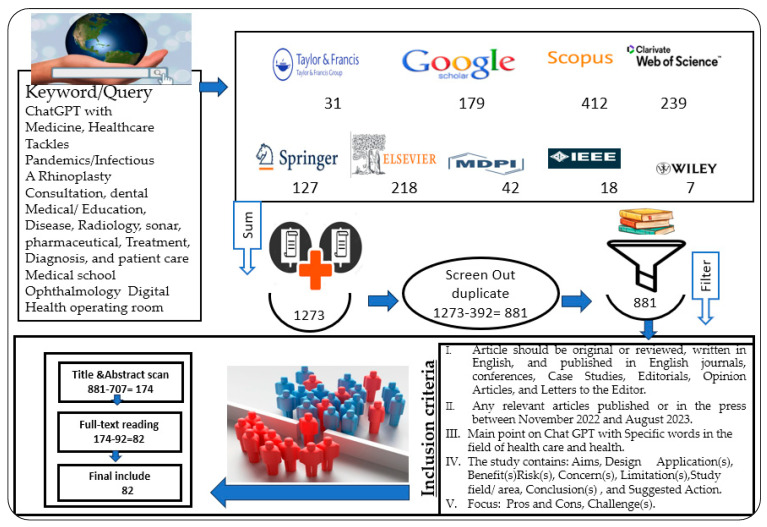
Flowchart for selecting studies with specific query and eligibility criteria.

The focus is on the pros and cons, challenges, as well as all the inclusion criteria shown in Figure 4.

### 2.2. Data Gathering Procedure

All included articles were reviewed, examined, and summarised in accordance with their basic classifications, then saved as Microsoft Word and Excel files to simplify the filtering process. For every piece, the authors read the complete text. We were able to develop the proposed taxonomy using a variety of highlights and comments on the surveyed works, as well as a running classification of all the articles. The remarks were recorded in either paper copy or electronic form, depending on the writing style of each contributor. After this, another procedure was conducted to characterise, describe, tabulate, and draw conclusions about the key findings. 

### 2.3. Research Questions

The selection of research questions is a critical step in shaping the purpose of the study and the anticipated results. Consequently, we have formulated the following research questions to align with the primary objective of our systematic literature review:

- RQ1: How does ChatGPT contribute to pandemic management and what specific advantages does it offer in disseminating critical information during health crises?
- RQ2: How is ChatGPT utilised in the field of dental practices and how does it enhance the overall patient experience in this context?
- RQ3: What challenges and ethical considerations are associated with the integration of ChatGPT into medical practices and healthcare settings?
- RQ4: What are the key components associated with work related to ChatGPT in medicine and healthcare and their contributions in ChatGPT applications in the field?

## 3. Results

The results of the initial query search, which yielded 1273 articles, are as follows: there are 31 articles from Taylor and Francis, 179 articles from Google Scholar, 412 articles from Scopus, 239 articles from Web of Science, 127 articles from Elsevier, 218 articles from Springer, 41 articles from MDPI, 18 articles from IEEE Xplore digital, and 7 articles from Wiley from between November 2022 and August 2023.

The papers were filtered according to the sequence that was adopted in this research and were divided into two categories: 1273 articles were published in the last eight months (November 2022 to August 2023) and 392 papers appeared in all nine databases or publishers, resulting in a total of 1547 papers. Following a comprehensive scan of the titles and abstracts of the papers, an additional 1273 papers were excluded. After the final full-text reading, 92 papers were excluded. The final set consisted of 82 papers, which were divided into eight major categories/groups as follows: G1: treatment and medicine are essential; G2: buildings and equipment play a crucial role; G3: parts of the human body and areas of disease; G4: patients; G5: citizens; G6: the focus is on radiology, pulse, and medical images; G7: revolves around doctors and nurses; and G8: encompasses tools, devices, and administration.

The first category (G1), which comprised 27 articles (32.93%), was focused on treatment and medicine. The second category (G2) consisted of two articles (2.44%) that were focused on buildings and equipment. The third category, known as G3, comprised 14 articles, accounting for 17.07% of the total. These articles focused on parts of the human body and areas affected by disease. The fourth category (G4), which comprised eight articles (9.76%), focused on patients. The fifth category (G5), which comprised three articles (3.66%), was citizens. The sixth category (G6) comprised four articles (4.88%) on cellular imaging, radiology, pulse, and medical images. The seventh category, known as G7, consisted of 17 articles, accounting for 20.73% of the total. This category focused on doctors and nurses. The eighth category, known as G8, consisted of seven articles, accounting for 8.54% of the total. This category focused on tools, devices, and administration.

### 3.1. RQ1: How Does ChatGPT Contribute to Pandemic Management and What Specific Advantages Does It Offer in Disseminating Critical Information during Health Crises?

ChatGPT plays a crucial role in pandemic management by aiding in the swift and accurate dissemination of critical information. Its natural language processing capabilities enable it to generate coherent responses, making it a valuable tool for healthcare organisations. Specific advantages include its ability to provide rapid updates, preventive measures, and medical guidelines to the public. ChatGPT assists in addressing queries and concerns, ensuring accurate information flow and timely interventions during health crises [5,6,7,8,9,42,43].

### 3.2. RQ2: How Is ChatGPT Utilised in the Field of Dental Practices, and How Does It Enhance the Overall Patient Experience in This Context?

ChatGPT is integrated into dental practices in order to empower dental assistants and improve the overall patient experience. It assists in the handling of patient inquiries, the provision of oral health tips, and the guidance of patients through postoperative care instructions. ChatGPT ensures standardised and accurate responses, thereby assisting patients in making informed decisions regarding their dental care. This AI integration revolutionises patient communication, appointment scheduling, and postoperative support in dental practices [8,11,12].

### 3.3. RQ3: What Challenges and Ethical Considerations Are Associated with the Integration of ChatGPT into Medical Practices and Healthcare Settings?

The integration of ChatGPT into medical practices and healthcare settings presents challenges and ethical considerations. One challenge is that the responses of ChatGPT are based on the data on which it was trained, which may result in biased or inaccurate information. Ethical concerns arise when content generated by AI is mistaken for the expertise of a human. The challenge lies in striking a balance between the capabilities of AI and the necessity for human judgement and expertise. Ensuring that AI does not replace human healthcare professionals entirely while leveraging its advantages is a significant ethical consideration [18,19,27,31,35,38,39,40,41]. Careful thought and responsible adoption are essential to mitigate potential risks and challenges.

### 3.4. RQ4: What Are the Key Components Associated with Work Related to ChatGPT in Medicine and Healthcare and Their Contributions in ChatGPT Applications in the Field?

The findings of our studies are summarised in Table 1, which includes information about the purpose of the study (in the “aim of study” column), the design and application (in the “design, application(s)” column), the benefits and risks (in the “benefit(s), risks” column), the summary of results for concerns and limitations (in the “concern(s), limitation(s)” column), the main outcomes, the type of study (in the “study field/area” column), and the main outcomes reported by each article in the conclusion section (in the “Suggested Action” column). Table 2 summarises the pros and cons or challenges of some of the papers that were exposed.

## 4. Challenges

In the field of healthcare and the medical field, refer to the difficult or complex issues, obstacles, or problems that healthcare professionals, researchers, organisations, and technologies face while providing medical care, conducting research, and addressing public health concerns. These challenges can arise due to various factors such as scientific advancements, technological limitations, ethical considerations, regulatory frameworks, economic constraints, patient expectations, and more.

**Figure 5:**
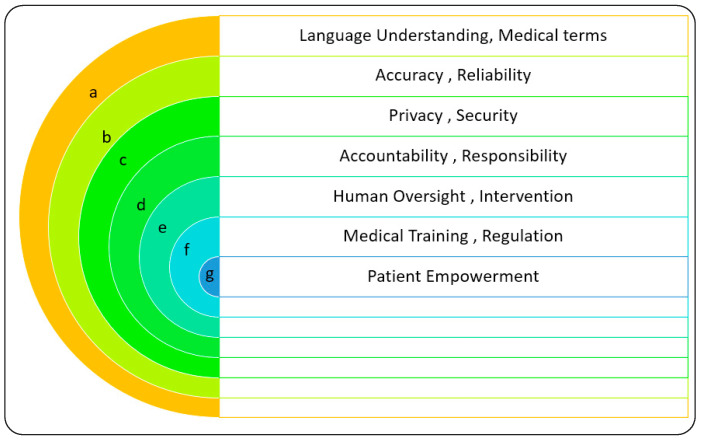
Challenges of healthcare and the medical fields.

In the context of healthcare and the medical field, challenges can encompass a wide range of issues, including but not limited to medical advancements, patient care, resource allocation, healthcare access, disease prevention and control, chronic disease management, healthcare costs, medical ethics, data privacy and security, protecting patient data and ensuring compliance with privacy regulations, interdisciplinary collaboration, patient education, and public health initiatives. In Figure 5, there were numerous challenges that Chatbot models such as GPT-3 could face in the field of healthcare and medicine.

### 4.1. Language Understanding and Medical Terms

Medical professionals frequently use specialised terminology and jargon that may be difficult for a general-purpose chatbot to understand. It is difficult to ensure that the chatbot understands medical terminology correctly.

*Importance:* this paper can illustrate the sophistication needed in natural language processing for medical contexts, underscoring the potential of advanced AI models to bridge communication gaps in healthcare.

*Benefits:* readers will discover the critical role of contextually aware AI in comprehending patient interactions, resulting in better patient support and care.

### 4.2. Accuracy and Reliability

Of utmost importance in healthcare is ensuring that the information provided by the chatbot is accurate and reliable. Medical information, which can be complex and critical, must be accurate to avoid misinformation and potential harm to patients.

*Importance:* this paper’s contribution to the ongoing conversation about the reliability of AI in clinical settings is highlighted, presenting the chatbot as a tool to augment, rather than replace, human judgement.

*Benefits:* readers can derive advantages from comprehending the significance of error-checking mechanisms and the ongoing updating of medical databases that AI systems must integrate.

### 4.3. Privacy and Security

Healthcare data are highly sensitive and subject to strict privacy regulations (like HIPAA in the United States). Chatbots need to adhere to these regulations and ensure that patient data are handled securely and confidentially.

*Importance:* the stress is on the advanced security protocols and compliance standards that AI systems must adhere to, as they are a cornerstone of healthcare technology. 

*Benefits:* this paper educates readers on the stringent data protection measures required for AI integration in healthcare, with an emphasis on the technology’s potential to maintain confidentiality.

### 4.4. Accountability and Responsibility

Decisions made by chatbots in healthcare settings can indeed have real-life consequences. Determining who is accountable for incorrect advice or recommendations provided by a chatbot can be a complex matter.

*Importance:* enriching the discourse on the role of AI in healthcare decision making by examining the legal and ethical frameworks that regulate AI.

*Benefits:* the survey can educate readers on the complex interaction between AI recommendations and human decision making, as well as the legal implications.

### 4.5. Human Oversight and Intervention

Although chatbots can assist in various healthcare tasks, there is a requirement for human oversight and intervention, particularly in critical situations. The challenge lies in balancing automation with the human touch.

*Importance:* the need for human experts to oversee AI systems is underlined, reinforcing the notion of AI as a supportive tool rather than a replacement.

*Benefits:* healthcare professionals can learn about the importance of their experience in supervising AI, ensuring patient safety and providing high-quality care. 

### 4.6. Medical Training and Regulation

Chatbots that provide medical advice may unintentionally bypass the regulatory frameworks established for traditional healthcare providers. It is crucial to ensure that chatbots are developed and used within established medical guidelines.

*Importance:* address the incorporation of AI within existing medical regulations, emphasising how the paper advances the conversation about regulatory adaptations for AI tools.

*Benefits:* this paper can help medical professionals understand the importance of regulatory compliance for AI, as well as encourage proactive engagement with technology.

### 4.7. Patient Empowerment

While chatbots can provide information, they should also encourage patients to consult qualified healthcare professionals for personalised advice. It is critical to strike a balance between empowerment and not substituting professional medical care.

*Importance:* it highlights the chatbot’s contribution to promoting well-informed patient decision making, underscoring the paper’s adherence to patient-centred care models.

*Benefits:* readers will appreciate how AI can provide information to patients while also understanding AI’s limitations in providing personalised medical advice.

## 5. Limitations and the Motivation

The limitations of these studies include a tendency to focus on theoretical potential rather than practical implementation, which leaves some real-world challenges unaddressed. Furthermore, significant concerns persist regarding the ethical and legal issues surrounding the roles, accuracy, and originality of AI. There exists a necessity for more comprehensive evaluations of content generated by AI and its impact on tasks performed by humans. Additionally, there is a potential risk of overreliance, which could result in a decrease in human critical thinking and involvement. Current studies frequently lack a thorough exploration of the broader implications, which necessitates adopting a more holistic approach to comprehending the complete scope and consequences of integrating AI, such as ChatGPT, into diverse fields.

The integration of ChatGPT in medicine and healthcare, though promising, does come with certain limitations. One key concern is the potential for inaccuracies in medical information provided by ChatGPT, as it lacks the ability to fully comprehend complex medical contexts. Serious medical errors could occur if patient queries are misinterpreted or incorrect diagnoses are generated. Furthermore, the reliance of ChatGPT on training data may introduce biases, which could potentially impact the quality and fairness of the provided information. Ethical challenges arise regarding patient data privacy and consent, as well as accountability for AI-generated medical content. The sharing of outdated or obsolete medical information could be a potential consequence of the lack of real-time updates and dynamic learning in ChatGPT. Lastly, there is a risk of overdependence on AI, which may diminish the role of healthcare professionals, thereby reducing critical thinking and human interaction in medical care.

The motivation for integrating ChatGPT with medicine and healthcare stems from the potential to improve patient care, medical research, and clinical decision-making processes. ChatGPT’s natural language processing capabilities provide a user-friendly interface for patients to seek medical information, resulting in increased patient engagement and empowerment. Its ability to analyse large amounts of medical literature assists healthcare professionals in staying up to date on the latest research and treatment options. ChatGPT can assist in the generation of accurate and concise medical documentation, thereby streamlining administrative tasks for healthcare providers. In addition, it holds promise in facilitating medical education, supporting remote consultations, and optimising clinical workflows. In general, the integration of ChatGPT with medicine and healthcare is in line with the objective of utilising AI technology to progress medical practices and improve patient outcomes.

## 6. Conclusions

As part of this investigation, a PRISMA-based systematic literature review was conducted. AI has the potential to transform many industries, including medicine and healthcare. Large language models, such as ChatGPT, have attracted attention for their ability to generate human-like text, revolutionising the healthcare landscape. This paper investigated ChatGPT’s applications in medicine, including pandemic management, surgical consultations such as cosmetic orthognathic surgery, dental practices, medical education, disease diagnosis, radiology and sonar imaging, pharmaceutical research, and treatment. The use of ChatGPT in disseminating critical information during pandemics and infectious diseases has showcased its ability to efficiently communicate essential updates and guidelines to the public. Furthermore, ChatGPT demonstrated its role as a virtual assistant in the context of cosmetic orthognathic surgery consultations. It provided standardised information and facilitated informed patient decisions. The integration of AI into dental practices has enhanced patient experiences by efficiently addressing inquiries and providing guidance.

The ability of ChatGPT to simplify complex medical concepts has enhanced medical education, leading to improved knowledge acquisition. It also displayed potential in disease diagnosis by offering differential diagnoses based on patient-reported symptoms and medical history. In the fields of cellular imaging, radiology, and sonar imaging, the descriptive capabilities of ChatGPT could be helpful for radiologists in the analysis of medical images and cellular imaging. Pharmaceutical research, benefiting from ChatGPT, was another realm that expedited research processes. It accomplished this by sifting through scientific literature and suggesting research directions. However, ChatGPT offers immense potential; however, it also has limitations that stem from its training data and ethical concerns that are related to its output. In conclusion, the integration of ChatGPT into medicine and healthcare holds vast transformative potential. It is crucial to responsibly utilise the capabilities of ChatGPT as technology advances, while also addressing its limitations and ethical considerations. The role of ChatGPT as a catalyst for innovation, advancement, and improved patient care within the medical landscape is underscored by this exploration.

Future work for ChatGPT in AI for healthcare includes the enhancement of diagnostic precision, the personalisation of care, the improvement of EHR integration, the assurance of regulatory compliance, the expansion of telemedicine capabilities, the advancement of medical education tools, and addressing ethical considerations in patient AI interactions.

## Figures and Tables

**Table 1:** The summary of related works includes the study’s goal, design, application(s), benefit(s), risk(s), concern(s), limitation(s), study field/area, conclusion(s), and suggested action for ChatGPT with medicine and healthcare.

No.	References	Aims, Design	Application(s), Benefit(s)	Risk(s), Concern(s), Limitation(s)	Study Field/Area and Categories (G)	Conclusion(s), Suggested Action
1	[51]	Evaluate ChatGPT’s medical applications via systematic review of articles.	Streamline tasks, improve care, decision making, communication in medicine.	Privacy, ethics, bias concerns; ChatGPT transforms medicine with potential for innovation. Review of limitations to other studies.	Medicine, G4.	ChatGPT’s medical potential is promising but faces challenges. Ethical, safety considerations vital for transforming healthcare with AI.
2	[13]	Study examines ChatGPT’s integration in education, research, and healthcare perspectives.	ChatGPT’s applications: text generation, literature analysis, image learning in education, healthcare, and research.	Ongoing ethical exploration is needed. Society’s AI impact requires scrutiny. Efficiency vs. bias trade-off. Risk of the digital divide widening.	Education, healthcare, research, G1.	Responsible AI use demands transparency, equity, reliability, and non-harm principles.
3	[44]	Examine ChatGPT’s utility in healthcare education, weigh pros and cons.	Enhances personalised learning, clinical reasoning, and skills development.	Benefits include interactive learning, but drawbacks like bias, data privacy persist.	Medical, dental, pharmacy, and public health, G1.	ChatGPT’s integration in healthcare education offers potential advantages. Further research required to address ethical, bias, and accuracy concerns.
4	[5]	Assess ChatGPT’s medical query accuracy and completeness for physicians.	ChatGPT as medical information source with substantial accuracy and potential.	ChatGPT’s medical accuracy is promising but not entirely reliable. Higher accuracy scores often contrasted with surprising errors. Limited sample size; potential selection and respondent biases. Patient-generated queries, complex cases not well-represented.	Medical, G4, G5.	ChatGPT shows potential as medical resource but requires validation and improvement.
5	[15]	Evaluate ChatGPT’s performance in medical education, content generation, and deception.	Medical education, skill practice, patient interaction simulation, content generation.	ChatGPT aids learning but raises ethical concerns. Can deceive professionals and educators. Risk/Classer A Double-Edged Sword.	Medical, G1, G4, G5.	ChatGPT’s potential in education is transformative yet raises ethical concerns.
6	[6]	Evaluate ChatGPT’s utility in healthcare education, research, and practice. Systematic Review.	Improved writing, efficient research, personalised learning, streamlined practice.	Promising applications but ethical. Accuracy concerns require cautious integration. Risk: Overzealous adoption of ChatGPT may overlook ethical limitations.	Healthcare Education, Research, Practice, G5, G7	Widespread LLM use is inevitable; ethical guidelines crucial. ChatGPT’s potential in healthcare must be carefully weighed against risks. Careful implementation with human expertise essential to avoid misuse and harm.
7	[6]	Assess ChatGPT’s utility and limitations in healthcare education, research, practice. Systematic Review.	Efficient research, personalised learning, streamlined practice, enhanced writing.	Promising potential. Ethical concerns warrant careful application and guidelines.	Healthcare Education, Research, Practice, G 5, G8.	Imminent LLM adoption, guided by guidelines, balances potential and risks.
8	[16]	Explore ChatGPT’s use in medical education perceptions and experiences.	ChatGPT can aid information collection, saving time and effort.	Study highlights ChatGPT’s potential but raises concerns. Further research is needed. Accuracy in extracting results.	Medical Education, G3, G4, G7.	Study offers insights: ChatGPT’s pros acknowledged, concerns raised, need further research for successful integration in medical education.
9	[7]	Investigate ChatGPT’s potential in simplifying medical reports for radiology, Radiology Reports (Case Study).	ChatGPT simplifies radiology reports; improves patient-centred care.	Initial study shows ChatGPT’s potential in medical report simplification. Small sample sizes (3 original reports, 15 experts). Use of fictitious radiology reports, not real patient data. Radiologist generating simplified reports is non-native English speaker. Original reports with unique German abbreviation (“Z.n.”) not in English. Quality assessed qualitatively; patients’ perspective not included.	Medical/Radiology Reports, G6.	Positive potential of LLMs for radiology report simplification; need for technical improvements and further research.
10	[52]	Analyse differences between human-written medical texts and ChatGPT-generated texts, develop detection methods.	Enhance trustworthy medical text generation, improve detection accuracy.	Limited scope, language proficiency, nuanced context understanding, potential bias.	Medical Texts, G5.	ChatGPT-generated medical texts differ from human-written ones, detection methods effective. Trustworthy application of large language models in medicine promoted.
11	[9]	Summarise ChatGPT’s role in medical education and healthcare literature, Hybrid Literature Review.	Insight into ChatGPT’s impact on medical education, research, writing.	Insight into ChatGPT’s impact on medical education, research, writing.	Presence G5, G6.	Review highlights ChatGPT’s medical role, urges criteria for co-authorship.
12	[8]	Review the use of ChatGPT in medical and dental research.	ChatGPT assists in academic paper search, summarisation, translation, and scientific writing.	AI aids data visualisation and comprehension, overcoming human limitations. Ethical concerns. Limited discussion. Need for regulation.	Medical/Dental Research, G7.	ChatGPT aids research but ethical concerns and limitations require examination.
13	[53]	Investigate ChatGPT’s healthcare applications via an interview.	Rapid, informative response generation; written content quality.	Ethical concerns. Potential limitations in healthcare utilisation.	Health Care, G1 G5, G6.	Not available.
14	[54]	Analyse ChatGPT’s use in healthcare, emphasising its status and potential. Taxonomy/Systematic Review.	Provides insights into ChatGPT’s medical applications, informs healthcare professionals.	Current ChatGPT performance limits clinical deployment. Specialised models preferred.	Healthcare G4, G5, G7, G8.	This study evaluates ChatGPT’s medical applications, emphasising insights and limitations. Clinical deployment remains unfeasible due to current performance.
15	[39]	Analyse ChatGPT’s AI applications, benefits, limitations, ethics in healthcare.	Medical research, diagnosis aid, education, patient assistance, updates.	Ethical, legal concerns. Copyright, transparency. Medico-legal complications.	Medicine, G4.	ChatGPT has healthcare applications, but ethical concerns and limitations need addressing.
16	[55]	Evaluate ChatGPT’s medical utility and accuracy in healthcare discourse.	Rapid response for medical queries, user-friendly interaction.	Generalised responses. Errors in medical answers. Limited data scope.	Healthcare/research, G2, G5, G6.	ChatGPT offers limited utility in healthcare, requiring fact-checking and awareness.
17	[56]	Discuss opportunities and risks of ChatGPT’s implementation in various fields.	Easy communication, potential for improving content quality.	Challenges of authenticity. Ethical concerns. Overreliance. Study is only two questions.	Medicine, science, academic publishing, G1, G4, G5, G6	Embrace AI, but thoughtfully, considering benefits and risks.
18	[57]	Evaluate ChatGPT’s potential as a medical chatbot, addressing concerns.	Enhancing healthcare access with technology, while raising ethical concerns.	Accuracy, transparency. Ethical issues, biases, safeguards. Potential obstacles in development.	Medical/ chatbots, G1, G5, G6, G8.	Balancing ChatGPT’s healthcare potential and concerns requires careful consideration, safeguards, and ongoing improvement.
19	[17]	Evaluate ChatGPT’s performance in medical question answering.	Assessing ChatGPT’s accuracy in medical exam questions.	Decreased accuracy with higher question difficulty. External information limitation. Difficulty dependency. Limited external information. Model accuracy. Question specificity.	Medical/ Licensing Exams, G1, G7.	ChatGPT shows promise as a medical education tool for answering questions with reasoning and context.
20	[40]	Evaluate ChatGPT’s role in medical research.	Enhances drug development, literature review, report improvement, personalised medicine, and more.	Potential accuracy, originality, integrity. Ethical concerns need addressing. Lack of specialisation. Need for further training. Ethical considerations. Risk of inaccuracies.	Medical/ Research, G4, G5.	ChatGPT offers transformative potential but requires addressing accuracy, integrity, and ethical considerations for clinical application.
21	[58]	Investigate the use of ChatGPT in expediting literature review articles creation, focusing on Digital Twin applications in healthcare. Literature Review.	Utilising ChatGPT for literature review accelerates knowledge compilation, easing academic efforts and focusing on research.	Paraphrased content showed significant matches using plagiarism detection tool. Raising concerns about originality. Content quality.	Healthcare/ Digital Twin, G1, G3, G4.	ChatGPT generated Digital Twin in healthcare articles. Low plagiarism in author-written text, high similarity in abstract paraphrases. AI accelerates knowledge expression, academic validity monitored through citations.
22	[59]	Explore ChatGPT’s healthcare applications, discuss limitations, and benefits.	Medical data analysis, chatbots, virtual assistants, language processing.	Data accuracy, privacy. Ethics, medical expertise. Accountability challenges	Healthcare, G6, G8.	ChatGPT has versatile healthcare applications but faces ethical, privacy, and accuracy concerns.
23	[60]	Examine AI impact on medical publishing ethics and guidelines.	AI-generated content, democratisation of knowledge dissemination, multi-language support.	Limitation: Misleading content, ethical concerns. Misinformation potential, unequal access to monetised AI-generated content.	Medical/ publishing, G3, G5, G6.	AI like ChatGPT can democratise knowledge, but ethical, accuracy, and access challenges need comprehensive consideration and guidelines.
24	[25]	Evaluate AI impact on academic writing integrity and learning enhancement.	Academic writing, learning enhancement, objective evaluation of AI-generated content.	Limited depth, errors, AI detection. Inappropriate for professional communication. Limits cheating advantage. Hinders learning and writing benefits. Constrains potential educational applications. Useful in medical imaging student learning enrichment.	Medical/ imaging, G6.	Conclusion: ChatGPT shows potential for learning enhancement but risks academic integrity and lacks depth for advanced subjects.
25	[61]	Explore ChatGPT’s capabilities for medical education and practice.	ChatGPT aids medical education, generates patient simulations, quizzes, research summaries, and promotes AI learning.	Occasional mistakes, hallucinations. Lacking proper prompting.	Medical education, G1, G4, G5, G8.	ChatGPT shows potential for medical education, research, but faces limitations and challenges. JMIR Medical Education is launching a theme issue on AI.
26	[26]	To explore the potential applications of ChatGPT in assisting researchers with tasks such as literature review, data analysis, hypothesis creation, and text generation.	Literature review, data analysis, hypothesis creation, text generation.	Plagiarism risk. Misleading results. Lack of context and nuance. Generalised perspective.	Medical research, G6, G8.	Scientific caution is needed in using ChatGPT due to plagiarism risks, misleading outcomes, lack of context, and AI limitations.
27	[62]	To examine the potential impact of large language models (LLM), specifically “ChatGPT”, on the nuclear medicine community and its reliability in generating nuclear medicine and molecular imaging-related content.	Text generation, collaborative tool	Outdated training knowledge (2021 cut-off). 34% accuracy in multiple-choice. Limited learning and improvement. Inadequate nuanced understanding.	Medicine/ nuclear, G6.	ChatGPT’s multiple-choice answering accuracy was 34%, surpassing random guessing. Improving training and learning capabilities is crucial.
28	[63]	To explore applications and limitations of the language model ChatGPT in healthcare.	Investigating ChatGPT’s potential in clinical practice, scientific production, reasoning, and education.	Complex medical reasoning. Ethical implications. Real-world performance uncertainty. High-level clinical expertise challenge.	Healthcare, Clinical, Research Scenarios, G5, G7	ChatGPT’s utilisation in healthcare requires cautiousness, considering its capabilities and ethical concerns.
29	[64]	To comprehensively review ChatGPT’s performance, applications, challenges, and future prospects.	Explore ChatGPT’s potential across various domains, anticipate future advancements, and guide research and development.	Bias and trust. Incomplete understanding. Ethical concerns. Limited context.	Mult, medical, G6.	Review identifies ChatGPT’s potential, applications, limitations, and suggests future improvements.
30	[65]	This study aims to examine the potential and limitations of ChatGPT in medical research and education, focusing on its applications and ethical considerations.	ChatGPT can support researchers in literature review, data analysis, hypothesis generation, and medical education. Its AI capabilities enable efficient information extraction and text generation, enhancing research and learning processes.	Caution required. Human input essential. Ethical exploration needed. Ongoing monitoring.	Clinical, translational medicine, G3, G6.	ChatGPT’s applications in scientific research must be approached cautiously, considering evolving limitations and human input, with focus on research ethics and integrity.
31	[28]	To explore the potential of Large Language Models (LLMs) like OpenAI’s ChatGPT in medical imaging, investigating their impact on radiology and healthcare.	LLMs enhance radiologists’ interpretation skills, facilitate patient–doctor communication, and optimise clinical workflows, potentially improving medical diagnosis and treatment planning.	Data quality and quantity affect LLM performance. Medical knowledge gaps can lead to incorrect information. Ethical concerns arise due to potential biases and sensitive content. Sole reliance on LLMs reduces human involvement and oversight.	Medical/ imaging, G6.	Large Language Models (LLMs), in medical imaging promise revolutionary impact with research and ethics.
32	[66]	Investigate ChatGPT’s role in medical education, exploring its applications, benefits, limitations, and challenges; Scoping Review.	ChatGPT aids automated scoring, personalised learning, case generation, research, content creation, and translation in medical education.	Limited reasoning ability beyond existing knowledge. Potential generation of incorrect information and biases. Undermining critical thinking skills. Ethical concerns, privacy issues, and potential for cheating.	Medical/ Education, G1, G5.	ChatGPT enhances medical education with personalised learning, yet its limitations, biases, and challenges warrant cautious implementation and evaluation.
33	[67]	This study examines if ChatGPT-4 can provide accurate and safe medical information to patients considering blepharoplasty.	ChatGPT-4 aids patient education, offers evidence-based information, and improves communication between medical professionals and patients.	Incomplete Understanding: ChatGPT may not fully comprehend complex medical nuances. Lack of Personalisation: Responses might lack customisation to individual patient needs. Misinterpretation: Generated information might not cover all potential variations or complications. Ethical Concern: Reliance on AI may reduce personal.	Medical/ Blepharoplasties, G1, G5, G7.	ChatGPT-4 shows potential in patient education for cosmetic surgery, offering accurate and clear information, but its limitations require consideration.
34	[12]	To explore the capabilities and applications of ChatGPT, a large language model developed by OpenAI.	Chatbots, language translation, text completion, question answering.	Contextual Understanding: ChatGPT might struggle with nuanced context understanding. Biases: The model can inadvertently generate biased content present in the training data. Inaccuracies: It may produce incorrect or misleading information due to limited fact-checking capabilities. Lack of Human Judgment: ChatGPT lacks the human ability to critically analyse or interpret complex situations.	The Use of Cybersecurity to Protect Medical Information, G1, G4, G5.	Not available.
35	[10]	Explore AI language model ChatGPT’s viability as a clinical assistant. Evaluate ChatGPT’s ability to provide informative and accurate responses during initial consultations about cosmetic orthognathic surgery.	ChatGPT can assist patients with medical queries.	Lack of personalised advice. Inability to provide detailed information. Limited scope for complex medical cases. Potential misinformation due to lack of medical expertise.	A Cosmetic orthognathic surgery consultation, G3, G4.	ChatGPT demonstrates potential in offering valuable medical information to patients, particularly when access to professionals is restricted. However, its limitations and scope should be further investigated for safe and effective use in healthcare.
36	[34]	To examine the impact of artificial intelligence (AI) on dental practice, particularly in CBCT data management, and explore the potential benefits and limitations.	AI-driven Cone-beam-computed tomography (CBCT) data management can revolutionise dental practice workflow by improving efficiency and accuracy. Segmentation automation aids treatment planning and patient communication, enhancing overall care.	High bias risk and inadequate sample sizes in studies may affect the accuracy. Generalisability of AI solutions. Potential errors and reliability concerns must be addressed.	Standard Medical Diagnostic/ dental, G3.	AI integration in dental practice, particularly CBCT data management, shows promise in enhancing efficiency, accuracy, and patient communication while facing bias and reliability challenges.
37	[35]	To explore the potential applications of ChatGPT, in managing and controlling infectious diseases, focusing on information dissemination, diagnosis, treatment, and research.	ChatGPT can enhance infectious disease management by providing accurate information, aiding diagnosis, suggesting treatment options, and supporting research efforts, ultimately improving patient care and public health.	Data Dependence: ChatGPT’s accuracy relies on the quality and quantity of training data. Medical Expertise: it lacks specialised medical knowledge, potentially leading to inaccurate advice. Ethical Considerations: generated content may propagate biases or sensitive information. Risk of Misuse: users might rely solely on AI-generated recommendations, bypassing medical professionals.	Tackles Pandemics/Infectious Disease, G4.	ChatGPT exhibits transformative potential in infectious disease management, though data reliance, medical accuracy, ethical concerns, and misuse risks require careful consideration.
38	[68]	To explore the integration of AI and algorithms to enhance physician workflow, maintain patient–physician rapport, and streamline administrative tasks.	AI can seamlessly assist doctors in clinical note generation, order selection, coding, history gathering, inbox filtering, and billing processes, improving efficiency and accuracy.	Seamless Integration: AI must seamlessly blend into physician workflows without disrupting decision making or creating pop-up warnings. Accuracy and Reliability: AI-generated content, like CPT codes, requires training for accuracy, and physician verification remains essential. Data Privacy: AI’s reliance on patient history and medical records necessitates stringent data privacy measures. Oversight and Dependency: Physicians must maintain oversight and avoid excessive reliance on AI-generated content, preserving professional judgment.	Medicine/ De-Tether the Physician, G2.	Not available.
39	[69]	To explore the implications of large language models (LLMs), particularly ChatGPT, for the field of academic paper authorship and authority, and to address the ethical concerns and challenges they introduce in health professions education (HPE).	Authorship and authority assessment, scholarly communication enhancement, technological advancement reflection.	Authenticity verification. Quality and nuance. Bias and objectivity. Loss of human connection.	Artificial scholarship: LLMs in health professions education research, G5, G7, G8.	Not available.
40	[70]	To assess ChatGPT’s accuracy and reproducibility in responding to patient queries about bariatric surgery.	ChatGPT serves as an information source for patient inquiries about bariatric surgery, aiding patient education and enhancing their understanding of the procedure.	Domain Specificity: ChatGPT may lack in-depth medical knowledge for complex questions Biased Responses: Generated responses might reflect biases present in training data. Lack of Contextual Understanding: Responses may not account for individual patient context or needs. Overreliance on Technology: Relying solely on ChatGPT can undermine personalised patient–doctor interactions.	Bariatric Surgery, G5, G4.	ChatGPT offers accurate responses for bariatric surgery inquiries. It is a valuable adjunct to patient education alongside healthcare professionals, fostering better outcomes and quality of life through technology integration.
41	[71]	Investigate ChatGPT’s behaviour, geolocation impact, and grammatical tuning, and address performance concerns.	Inform ChatGPT’s reliable use in education and medical assessments.	No geolocation evidence. Unclear grammatical impact. Assessment comparability issues. Response comparison challenges.	Medical Licensing/Certification Examinations.	Not available.
42	[18]	Explore ethical considerations in integrating AI applications into medical education, identifying concerns and proposing recommendations.	Integration in medical education for interactive learning. Benefits include personalised instruction and improved understanding.	Bias in AI-generated content. Reduced human interaction and empathy. Ethical dilemmas in AI-driven decision making. Privacy concerns with student data.	Medical/Education Biomedical Ethical Aspects. G3.	Integration of AI in medical education offers enhanced learning but demands a robust ethical framework, iteratively updated for evolving advancements and user input.
43	[72]	To investigate whether the use of ChatGPT technology can enhance communication in healthcare settings, leading to improved patient care and outcomes.	To explores the potential application of ChatGPT technology to address communication challenges in hospitals. By generating clear and understandable medical information, ChatGPT can bridge the communication gap between healthcare providers and patients. This could result in improved patient understanding, reduced miscommunication, and enhanced patient care quality.	The study acknowledges that, while ChatGPT and other AI-generated content have the potential to improve healthcare communication, they should not be seen as a complete replacement for human interaction. The limitations of AI, such as lack of empathy and context understanding, should be considered when implementing such technology. The study does not delve deeply into the technical challenges and potential biases that AI-generated content may carry.	Communication in hospitals, G4, G7.	ChatGPT aids hospitals in enhancing patient care and communication efficiency.
44	[24]	Evaluate ChatGPT’s reliability in diagnosing diseases and treating patients.	Preliminary medical assessments; quick information for users seeking advice.	Variable response quality. Inaccuracies in treatment recommendations, ethical concerns.	Diagnosis and patient care, G3, G4, G7.	ChatGPT’s case responses need improvement; users require expertise for interpretation.
45	[73]	To assess ChatGPT’s abilities in generating ophthalmic discharge summaries and operative notes.	ChatGPT can potentially aid in creating accurate and rapid ophthalmic discharge summaries.	The generated content might include generic text and disclaimers. The quality of responses varies based on the quality of inputs. Detailed operative notes require significant tuning for accuracy. ChatGPT’s responses may still contain factual inaccuracies that need correction.	Ophthalmology, G3.	ChatGPT’s performance in ophthalmic notes is promising, rapid, and impactful with focused training and human verification.
46	[36]	To assess healthcare workers’ knowledge, attitudes, and intended practices towards ChatGPT in Saudi Arabia.	ChatGPT can be used to support medical decision making, patient support, literature appraisal, research assistance, and enhance healthcare systems.	Concerns about credibility and source of information from AI Chatbots. Reliability and accuracy issues raise doubts about diagnostic and treatment use. AI chatbots should not replace human expert oversight for critical healthcare decisions. Future research needed to evaluate clinical outcomes and benchmark AI chatbot performance.	Digital Health, G4, G5.	This study highlights ChatGPT’s potential benefits in healthcare but concerns about accuracy and reliability persist. Trustworthy implementation requires addressing these issues.
47	[74]	To evaluate ChatGPT’s performance in comprehending complex surgical clinical data and its implications for surgical education.	Assessing ChatGPT (GPT-3.5 and GPT-4) in understanding surgical data for improved education and training.	Limited to Korean general surgery board exams, may not generalise to other medical fields. Performance improvement in GPT-4 may not eliminate all errors or replace human expertise. Evaluation does not consider nuances, real-time clinical context, or interaction dynamics. Ethical considerations on reliance on AI in critical healthcare decision making remain.	Operating room, G1, G3.	ChatGPT, especially GPT-4, excels in comprehending surgical data. Achieving 76.4% accuracy on the board exam, its potential must be combined with human expertise.
48	[19]	To assess ChatGPT’s factual medical knowledge by comparing its performance with medical students in a progress test.	ChatGPT’s AI offers easy medical knowledge access. It aids medical education and testing.	Limited contextual understanding due to lack of context. Inaccurate responses. Overreliance on text. Lack of human expertise.	Medical school, G3, G4, G7, G8.	ChatGPT accurately answered most MCQs in Progress Test Medicine, surpassing students’ performance in years 1–3, comparable to latter-stage students.
49	[20]	To explore the utility and accuracy of using ChatGPT-3, an AI language model, in understanding and discussing complex psychiatric diagnoses such as catatonia.	It investigates the potential of ChatGPT-3 as a tool for medical professionals to assist in understanding and discussing complex psychiatric diagnoses. It demonstrates the feasibility of using AI in medical research and education.	It relies on the accuracy of ChatGPT-3’s responses, which may not always be reliable. The study’s findings are based on the knowledge and expertise of the author and the neuropsychiatrist, which could introduce subjective biases. The accuracy of ChatGPT-3’s responses may vary depending on the quality of the prompt provided. It does not account for potential errors or limitations of ChatGPT-3’s training data.	Medical Education, G1, G7.	Not available.
50	[75]	To compare ChatGPT’s performance in answering medical questions with medical students’ performance in a progress test.	ChatGPT can aid medical education by providing factual knowledge. It offers quick access to information and can complement teaching materials.	ChatGPT’s responses might lack depth and nuanced understanding. Lack of Critical Thinking: ChatGPT may not engage in critical thinking or provide reasoning behind answers. Reliability: Reliance on AI might undermine the development of essential critical thinking skills in medical students. Lack of Clinical Judgment: ChatGPT cannot replace the clinical judgment and real-world experience of medical professionals.	Medical education, clinical management, G1.	ChatGPT is a valuable aid in medical education, research, and clinical management but not a human replacement. Despite limitations, AI’s rapid progress can enhance medical practices if embraced thoughtfully.
51	[29]	To explore the potential application of ChatGPT as a medical assistant in Mandarin-Chinese-speaking outpatient clinics, aiming to enhance patient satisfaction and communication.	ChatGPT enhances clinic communication, aids patient satisfaction, excels in exams, and improves interactions in non-English medical settings.	Optimisation needed. Integration challenges. Guidelines development. Clinical trials and approval.	Outpatient clinic settings, G4.	Not available.
52	[30]	To explore the role of AI in scientific article writing, particularly editorials, and its potential impact on rheumatologists.	AI, like ChatGPT, can aid rheumatologists in writing, improving efficiency. AI’s growing role in medicine, including image analysis.	Ethical concerns regarding AI dominance must be addressed. AI may replace human involvement in writing, impacting expertise and originality.	Rheumatologist + medical writing, G6.	AI offers scientific progress but caution is needed to avoid shallow work and hindered education. Beware AI dominance.
53	[76]	To evaluate the accuracy and effectiveness of using ChatGPT for simulating standardised patients (SP) in medical training.	ChatGPT is utilised to simulate patient interactions, saving time, resources, and eliminating complex preparation steps. Offers intelligent, colloquial, and accurate responses, potentially enhancing medical training efficiency.	Senior physician evaluation may introduce subjectivity. ChatGPT’s responses might not fully capture the nuances of real patient interactions. Its generalisability might be limited by the specific cases and physician preferences.	Clinical training, G4	Not available.
54	[77]	To evaluate the accuracy and effectiveness of using ChatGPT for simulating standardised patients (SP) in medical training.	ChatGPT is utilised to simulate patient interactions, saving time, resources, and eliminating complex preparation steps. Offers intelligent, colloquial, and accurate responses, potentially enhancing medical training efficiency.	Senior physician evaluation may introduce subjectivity. ChatGPT’s responses might not fully capture the nuances of real patient interactions. Generalisability might be limited by the specific cases and physician preferences.	Education, healthcare, research, G1.	Not available.
55	[21]	To evaluate the performance, potential applications, benefits, and limitations of ChatGPT in medical practice, education, and research.	ChatGPT demonstrates proficiency in medical exams and academic writing, potentially aiding medical education, research, and patient–provider communication.	ChatGPT’s handling of harmful content, misinformation, and plagiarism is unclear. Complex medical tasks and understanding human anatomy remain challenging. In radiological reports, accuracy and completeness can be concerns.	Medical practice + education + research, G1, G7.	ChatGPT holds potential for medical practice, education, and research but requires refinements before widespread use. Human judgment remains crucial despite its sophistication.
56	[78]	It is examining the necessity of traditional ethics education in healthcare training, given the capabilities of ChatGPT and other large language models (LLMs).	ChatGPT and LLMs can assist in fostering ethics competencies among future clinicians, aligning with bioethics education goals.	While ChatGPT and LLMs contribute to ethics education. They are not a complete replacement for human-led teaching due to inherent limitations.	Healthcare+ Ethics, G6, G7, G8.	Considering strengths and limitations, ChatGPT can be an adjunctive tool for ethics education in healthcare training, accounting for evolving technology.
57	[37]	To determine if ChatGPT can support multidisciplinary tumour board in breast cancer therapy planning.	ChatGPT’s application in aiding breast cancer therapy decisions; benefits include efficiency and broader information access.	Lack of Specificity: ChatGPT mostly offers general advice, lacking precision in therapy recommendations. Misidentification: Incorrectly identifying treatment needs, such as Her2 1+ and 2+ patients. Complex Case: Struggles with complex cases, mislabelling endocrine therapy as hormonal treatment. Inadequate Depth: Inability to provide comprehensive, patient-specific recommendations.	Cancer cases, G3.	Artificial intelligence aids personalised therapy; ChatGPT’s potential in clinical medicine is promising, but it lacks specific recommendations for primary breast cancer patients.
58	[22]	The aim of this study was to assess the performance of ChatGPT models in higher specialty training for neurology and neuroscience, particularly in the context of the UK medical education system.	It demonstrates the potential application of ChatGPT models in medical education, specifically in the field of neurology and neuroscience. It highlights their ability to perform at or above passing thresholds in specialised medical examinations, offering a tool for enhancing medical training and practice.	Limited specialty focus. Accuracy threshold. Clinical context.	Medical + Neurology, G7, G8.	ChatGPT-4’s progress showcases AI’s promise in medical education, but close collaboration is vital for sustained relevance and reliability in healthcare.
59	[79]	Evaluate ChatGPT’s performance in answering patients’ gastrointestinal health questions.	ChatGPT assists patients with health inquiries, potentially enhancing information accessibility in healthcare.	Question bias. Subjective grading. Expert selection. No comparisons.	Gastrointestinal Health, G3, G5.	ChatGPT’s potential in health info provision exists, but development and source quality improvement are necessary.
60	[80]	Investigate ChatGPT’s potential applications, especially in pharmacovigilance.	ChatGPT transforms human–machine interactions, offering innovative solutions, such as enhancing pharmacovigilance processes.	Potential for biased or incorrect outputs. Limited understanding of context and nuance. Dependency on input quality and relevance. Ethical concerns in sensitive domains.	Pharmacovigilance, G3, G4, G5.	Not available.
61	[38]	Evaluate the impact of AI, particularly ChatGPT, in the field of surgery, considering both its benefits and potential harms.	AI, including ChatGPT, can enhance surgical outcomes, diagnostics, and patient experiences. It offers efficiency and precision in surgical treatments.	Risk of inappropriate interventions by nonmedical individuals. Concerns over data privacy and security. Ethical challenges in patient data usage. Possibility of undermining medical professionals’ roles.	Intelligence, Surgery, G3, G6.	The growing influence of AI in surgery demands ethical contemplation.

**Table 2:** Summary of associated works includes all fields of study, study aim, pros, and cons or challenges.

No.	References	Field of Study	Study Aim	Pros	Cons, Challenge(s)
1	[38]	Surgery, Implications, Ethical Considerations.	Assess AI’s Impact on Surgery.	Improved Surgical Efficiency. Enhanced Diagnostic Capabilities. Refinement of Intra-operative Techniques. Long-term Complication Reduction. Advancements in Patient Experiences.	Potential Patient Harm. Undermining Medical Provider Roles. Layman Misuse and Interventions. Safety and Ethical Concerns. Patient Data Vulnerability. This study aims to evaluate the impact of AI in surgery, highlighting both its potential benefits and drawbacks.
2	[23]	Medical, education.	Integrate ChatGPT into Medical Education.	Clinical Reasoning Enhancement. Simplified Explanations and Mnemonics. Effective Practice Question Assistance. Improved Patient Communication Skills. Enhanced Differential Diagnosis Skills. AI-Generated Learning Reinforcement.	Ethical Concerns and Discussions. Variable Reliability and Accuracy. Reduced Human Interaction Impact. Technical Glitches Disruption Potential. Depersonalised Learning Experience Risk. Contextual Understanding Limitations.
3	[6]	Healthcare Education, Research, Practice.	To assess the utility of ChatGPT in healthcare education, research, and practice, highlighting its potential advantages and limitations.	Improved Scientific Writing. Enhanced Research Equity and Versatility. Efficient Healthcare Data Analysis. Code Generation for Research. Streamlined. Healthcare Workflow. Personalised Medicine Application. Improved Health Literacy. Enhanced Health Care Education.	Incorrect Citations. Ethical Concerns. Copyright and Legal Issues. Transparency Challenges. Risk of Bias and Plagiarism. Inaccurate or Hallucinatory Content. Limited Knowledge Base. Incorrect Citations. Cybersecurity Vulnerabilities. Potential for Misinformation.
4	[81]	medicine, ChatGPT.	Questioning AI, Role in Medicine.	Enhancing Efficiency Over Time. Technological Advancement. Potential Promising Applications. Evolution of AI Capabilities. Enhancing Efficiency Over Time.	Lack of Clear Purpose. Captivity to Trends and Fashions. Uncertainty About AI Maturity. Ethical and Qualification Concerns. Lack of Clear Purpose. Need for Evidence and Progress.
5	[41]	Healthcare.	Explore ChatGPT’s Role in Medicine.	Technological Innovation in Medicine. Enhanced Clinical Practice Guidelines. Evidence-Based Approach. Access to Personalised Insights. Support for Healthcare Professionals. Efficient Data-Driven Insights. Reduced Research Effort. Accelerated Guideline Creation.	Not a Substitute for Professionals. Need for Human Expertise. Potential Ethical Considerations.
6	[31]	Medicine.	Explore AI’s Impact on Paediatric Research.	Revolutionising Medicine with AI. Improved Clinical Decision Making. Enhanced Medical Education. Accelerated Drug Development. Better Research Outcomes. Advancement in AI Language Models.	Bias and Fairness Concerns. Safety and Security Issues. Overreliance on Technology. Ethical Considerations. Potential Negative Effects.
7	[32]	Medical Imaging, Radiologist.	Aims to explore the challenges faced in communicating radiation risks and benefits of radiological examinations, especially in cases involving vulnerable groups like pregnant women and children.	Promoting Radiation Protection. Justification and Optimisation of Exams. Confident Communication with Physicians. Enhanced Patient Informed Decision Making. Focus on Pregnant Women, Childbearing Age, Children. Building Trust and Reassurance. Patient-Centred Care.	Uncomfortable Patient Discussions. Challenges with Pregnant Women, Children. Addressing Radiation Risks. Difficulty in Communicating Benefits. Overcoming Fear and Misconceptions. Impact of Personal Trauma on Decisions.
8	[82]	medical examination, records, Chinese education.	To assess ChatGPT’s performance in understanding Chinese medical knowledge, its potential as an electronic health infrastructure, and its ability to improve medical tasks and interactions, while acknowledging challenges related to hallucinations and ethical considerations.	Chinese Medical Knowledge Assessment. Electronic Health Infrastructure Potential. Performance in Medical Exams and Records. Improved Accuracy with GPT-4. High Verbal Fluency. Logical Coherence in Discharge Summaries. Positive Human-Computer Interaction. GPT-4’s Advancements over GPT-3.5.	Hallucination Challenges. Legal and Ethical Concerns.
9	[83]	Infectious Disease.	This study aims to evaluate the potential utilisation of ChatGPT in clinical practice and scientific research of infectious diseases, along with discussing relevant social and ethical implications.	Humanistic AI Interaction. Rapid User Adoption. Potential Clinical and Research Use. Enhanced Disease Management Ideas. Quick Processing of Commands. Wide Public Engagement.	Ethical and Social Concerns. Privacy and Data Security Risks.
10	[84]	Medical, Test (Turing).	To evaluate the feasibility of using AI-based chatbots like ChatGPT for patient–provider communication, focusing on distinguishing responses, patient trust, and implications for healthcare interactions.	Chatbot Use for Patient–Provider Communication. Potential for Answering Low-Risk Health Questions. Patient Trust in Chatbot Functions. Study on Chatbot Interaction in Healthcare. Insights into Patient–Chatbot Perception.	Challenges in Distinguishing Responses. Limitations in Correct Source Identification. Variability in Patient Trust Levels Implications of Health-Related Complexity.
11	[85]	Medical applications.	Promoting Sustainable Practices in Medical 5G Communication.	Improved Patient Monitoring. Enhanced Care Co-ordination. Early Disease Detection. Patient Empowerment. Better Healthcare and Outcomes. Solar-Powered Emergency Backup. Utilisation of AI (ChatGPT). Climate Change Awareness.	Local Variability in Design. Power Supply Challenges. Dependence on Resources.
12	[86]	Patient Outcomes, Healthcare.	To investigate the potential applications of humanoid robots in the medical industry, considering their role during the COVID-19 pandemic and future possibilities, while emphasising the irreplaceable importance of human healthcare professionals and the complementary nature of robotics.	Assistance During Pandemic Situations. Constant Developments in Humanoid Robotics. Versatile Use in Various Industries. Potential Role in Medical Sector. Future Applications in Healthcare. Complementing Healthcare Initiatives.	Indispensable Role of Human Professionals. Limitations in Knowledge and Empathy. Need for Human Critical Judgment. Robots as Complementary, Not Full Replacements.
13	[87]	Medical.	Evaluating AI as Collaborative Research Partners.	Advanced Technological Support. Effective Knowledge Creation. Identifying Unseen Data Relationships. Synthesising and Explaining Information. Compliance with ICMJE Recommendations. Potential for AI as Co-Authors. Enhanced Academic Endeavours.	Ethical and Attribution Concerns Human Oversight and Responsibility.
14	[88]	Medicine, History.	To explore the importance of simplifying operations and creating user-friendly interfaces in AI-based medical applications, drawing insights from the success of ChatGPT and its impact on user adoption and clinical practice.	Improved Clinical Practice Applications. Increased AI Algorithm Development. Potential for Clinically Used Products. Lessons from ChatGPT’s Popularity. Emphasis on User-Friendly Interfaces. Simplification of Operations.	Complexity of AI Integration. Technical and Usability Challenges.
15	[89]	medical, education.	The aim of this specific aspect of the study is to critically analyse the manuscript and offer valuable feedback to improve its content, quality, and overall presentation.	Convenient Use of AI Models. Rapid and Time-Efficient Medical Writing. Assistance in Literature Searches and Draft Creation. Potential to Assist Medical Education and Clinical Decision Making. Potential for Quick Content Generation in Research.	Lack of Critical Thinking and Redundant Information. Potential for Cheating in Education. Erosion of Students’ Original Idea Generation. Accountability and Ethical Concerns. Medicolegal and Copyright Issues. Methodological Biases and Inaccuracies. Limited Access to Updated Training Data. Dependence on Restricted Databases. Limited Real-Time Information Extraction Capability. Lack of Clinical Reasoning and Critical Thinking in Content. Need for Human Oversight and Policies.
16	[11]	Dental assistant, Nurse.	To explores AI’s impact on dental assistants and nurses in orthodontic practices, examining evolving treatment workflows.	AI enhances treatment precision and personalisation. Automation of assessments saves time. AI can assist in identifying treatment progress. Potential for improved patient engagement. New roles for dental assistants and nurses. Can streamline administrative tasks. May lead to more efficient and effective care.	Raises ethical and legal concerns. Dependence on AI may reduce critical thinking. Patient trust in AI may vary. Implementation costs and training needed. Privacy and security risks with patient data. Potential for job displacement. Need for ongoing AI regulation and oversight.
17	[33]	ultrasound image guidance.	To assess the potential of using the Segment Anything Model (SAM) for intelligent ultrasound image guidance. It explores the application of SAM in accurately segmenting ultrasound images and discusses its potential contribution to a framework for autonomous and universal ultrasound image guidance.	Improved accuracy in ultrasound image segmentation. SAM could enhance the precision of medical procedures. Potential for reducing human error in image guidance. Advances in natural language processing (ChatGPT) and image segmentation (SAM) benefit medical practice. Universal ultrasound image guidance could improve accessibility to quality healthcare. Automation could lead to more efficient medical procedures.	Dependence on AI algorithms introduces a level of uncertainty. Implementation may require significant computational resources. Ethical and legal concerns regarding patient safety and consent. Need for ongoing training and validation of AI models. Potential for misinterpretation or errors in AI-generated guidance. Regulatory challenges related to AI-driven medical applications. Patient trust in AI-driven medical procedures may vary.
18	[90]	Health records.	Evaluate electronic medical records. (EMRs) foundation. models.	Potential for improved patient care and hospital operations.	Limited understanding of capabilities, small datasets, unclear usefulness to health systems.
19	[91]	Rheumatology.	Explore ChatGPT’s potential in rheumatology.	Rapid spread, versatile applications, efficient text generation.	Questions about convenience. Societal implications. Concerns about previous algorithms.
20	[40]	Medical Research.	Evaluate ChatGPT’s impact on medical research.	Potential for innovative research. Efficiency in literature review writing. Assistance in drug development. Improvement in medical report quality. Facilitation of data analysis. Personalised medicine applications.	Accuracy and reliability concerns. Potential lack of originality. Ethical considerations. Academic integrity challenges. Need for thorough validation. Limited studies and evidence.
21	[92]	Clinical Practice.	Explore ChatGPT’s applications and implications in clinical practice.	Accurate differential diagnosis lists. Enhanced clinical decision making. Improved clinical decision support. Reliable disease information. Efficient medical documentation. Potential for real-time monitoring. Precision medicine possibilities. Integration with. healthcare systems.	Ethical and legal concerns. Accuracy and reliability issues. Potential for misinformation. Dependence on AI reliability. Loss of human touch in care. Limited human judgment. Privacy and data security risks.
22	[93]	Bioethics.	Explore bioethical implications of ChatGPT.	Raises awareness of ethical concerns. Examines parallel with medical AI. Addresses data ownership and consent. Considers data representativeness. Highlights privacy challenges. Explores AI’s impact on informed consent. Addresses risks of medical deepfakes. Discusses equitable access issues. Considers environmental implications. Empowers patients and knowledge democratisation.	Uncertainty in social implications. Complex ethical dilemmas. Potential for misinformation. Ethical risks in AI development. Challenges of AI dominance races. Need for updated ethical frameworks.

## Data Availability

Not applicable.

## Supplementary Materials

The following supporting information can be downloaded at: https://www.mdpi.com/article/10.3390/diagnostics14010109/s1, Figure S1: PRISMA 2020 flow diagram for updated systematic reviews which included searches of databases and registers only; File S1: PRISMA 2020 checklist.

## References

[1] Agathokleous E., Saitanis C.J., Fang C., Yu Z. (2023). Use of ChatGPT: What Does It Mean for Biology and Environmental Science?. Sci. Total Environ..

[2] McGowan A., Gui Y., Dobbs M., Shuster S., Cotter M., Selloni A., Goodman M., Srivastava A., Cecchi G.A., Corcoran C.M. (2023). ChatGPT and Bard Exhibit Spontaneous Citation Fabrication during Psychiatry Literature Search. Psychiatry Res..

[3] Choudhary O.P. (2023). Priyanka ChatGPT in Travel Medicine: A Friend or Foe?. Travel Med. Infect. Dis..

[4] Kocoń J., Cichecki I., Kaszyca O., Kochanek M., Szydło D., Baran J., Bielaniewicz J., Gruza M., Janz A., Kanclerz K. (2023). ChatGPT: Jack of All Trades, Master of None. Inf. Fusion.

[5] Mueen Sahib T., Younis H.A., Mohammed A.O., Ali A.H., Salisu S., Noore A.A., Hayder I.M., Shahid M. (2023). ChatGPT in Waste Management: Is it a Profitable. Mesopotamian J. Big Data.

[6] Sallam M. (2023). ChatGPT Utility in Healthcare Education, Research, and Practice: Systematic Review on the Promising Perspectives and Valid Concerns. Healthcare.

[7] Jeblick K., Schachtner B., Dexl J., Mittermeier A., Stüber A.T., Topalis J., Weber T., Wesp P., Sabel B., Ricke J. (2022). ChatGPT Makes Medicine Easy to Swallow: An Exploratory Case Study on Simplified Radiology Reports. Eur. Radiol..

[8] Fatani B. (2023). ChatGPT for Future Medical and Dental Research. Cureus.

[9] Temsah O., Khan S.A., Chaiah Y., Senjab A., Alhasan K., Jamal A., Aljamaan F., Malki K.H., Halwani R., Al-Tawfiq J.A. (2023). Overview of Early ChatGPT’s Presence in Medical Literature: Insights from a Hybrid Literature Review by ChatGPT and Human Experts. Cureus.

[10] Xie Y., Seth I., Hunter-smith D.J., Rozen W.M., Ross R., Lee M. (2023). Aesthetic Surgery Advice and Counseling from Artificial Intelligence: A Rhinoplasty Consultation with ChatGPT. Aesthetic Plast. Surg..

[11] Surovkov J., Strunga M., Lifkov M., Thurzo A. (2023). The New Role of the Dental Assistant and Nurse in the Age of Advanced Artificial Intelligence in Telehealth Orthodontic Care with Dental Monitoring: Preliminary Report. Appl. Sci..

[12] Mijwil M., Aljanabi M., Ali A.H. (2023). ChatGPT: Exploring the Role of Cybersecurity in the Protection of Medical Information. Mesopotamian J. Cyber Secur..

[13] Hosseini M., Gao C.A., Liebovitz D., Carvalho A., Ahmad F.S., Luo Y., MacDonald N., Holmes A.K. (2023). An Exploratory Survey about Using ChatGPT in Education, Healthcare, and Research. PLoS ONE.

[14] Mohammed A.O., Salisu S.A., Younis H., Salman A.M., Sahib T.M., Akhtom D., Hayder I.M. (2023). ChatGPT Revisited: Using ChatGPT-4 for Finding References and Editing Language in Medical Scientific Articles. https://ssrn.com/abstract=4621581.

[15] Khairatun H.U., Miftahul A.M. (2023). ChatGPT and Medical Education: A Double-Edged Sword. J. Pedagog. Educ. Sci..

[16] Abouammoh N., Alhasan K.A., Raina R., Children A., Aljamaan F. (2023). Exploring Perceptions and Experiences of ChatGPT in Medical Education: A Qualitative Study Among Medical College Faculty and Students in Saudi Arabia Original Research: Exploring Perceptions and Experiences of ChatGPT in Medical Education: A Qualitativ. Cold Spring Harb. Lab..

[17] Gilson A., Safranek C.W., Huang T., Socrates V., Chi L., Taylor R.A., Chartash D. (2023). How Does ChatGPT Perform on the United States Medical Licensing Examination? The Implications of Large Language Models for Medical Education and Knowledge Assessment. JMIR Med. Educ..

[18] Busch F., Adams L.C., Bressem K.K. (2023). Biomedical Ethical Aspects Towards the Implementation of Artificial Intelligence in Medical Education in Medical Education. Med. Sci. Educ..

[19] Friederichs H., Friederichs W.J., März M., Friederichs H., Friederichs W.J., Chatgpt M.M., Friederichs H., Friederichs W.J. (2023). ChatGPT in Medical School: How Successful Is AI in Progress Testing? ChatGPT in Medical School: How Successful Is AI in Progress Testing?. Med. Educ. Online.

[20] Grabb D. (2023). ChatGPT in Medical Education: A Paradigm Shift or a Dangerous Tool?. Acad. Psychiatry.

[21] Sedaghat S. (2023). Early Applications of ChatGPT in Medical Practice, Education and Research. Clin. Med..

[22] Giannos P. (2023). Evaluating the Limits of AI in Medical Specialisation: ChatGPT’s Performance on the UK Neurology Specialty Certificate Examination. BMJ Neurol. Open.

[23] Guo A.A., Li J. (2023). Harnessing the Power of ChatGPT in Medical Education. Med. Teach..

[24] Huh S. (2023). Can We Trust AI Chatbots’ Answers about Disease Diagnosis and Patient Care?. J. Korean Med. Assoc..

[25] Currie G., Singh C., Nelson T., Nabasenja C., Al-Hayek Y., Spuur K. (2023). ChatGPT in Medical Imaging Higher Education. Radiography.

[26] Dahmen J., Kayaalp M.E., Ollivier M., Pareek A., Hirschmann M.T., Karlsson J., Winkler P.W. (2023). Artificial Intelligence Bot ChatGPT in Medical Research: The Potential Game Changer as a Double-Edged Sword. Knee Surg. Sports Traumatol. Arthrosc..

[27] Mohammed O., Thaeer M.S., Israa M.H., Sani S., Misbah S. (2023). ChatGPT Evaluation: Can It Replace Grammarly and Quillbot Tools?. Br. J. Appl. Linguistics.

[28] Yang J., Li H.B., Wei D. (2023). The Impact of ChatGPT and LLMs on Medical Imaging Stakeholders: Perspectives and Use Cases. arXiv.

[29] Zhu Z., Ying Y., Zhu J., Wu H. (2023). ChatGPT’s Potential Role in Non-English-Speaking Outpatient Clinic Settings. Digit. Health.

[30] Verhoeven F., Wendling D., Prati C. (2023). ChatGPT: When Artificial Intelligence Replaces the Rheumatologist in Medical Writing. Ann. Rheum. Dis..

[31] Corsello A., Santangelo A. (2023). May Artificial Intelligence Influence Future Pediatric Research?—The Case of ChatGPT. Children.

[32] Pozzessere C. (2023). Optimizing Communication of Radiation Exposure in Medical Imaging, the Radiologist Challenge. Tomography.

[33] Ning G., Liang H., Jiang Z., Zhang H., Liao H. (2023). The Potential of “Segment Anything” (SAM) for Universal Intelligent Ultrasound Image Guidance. Biosci. Trends.

[34] Strunga M., Thurzo A., Surovkov J., Lifkov M., Tom J. (2023). AI-Assisted CBCT Data Management in Modern Dental Practice: Benefits, Limitations and Innovations. Electronics.

[35] Pratim P., Poulami R. (2023). AI Tackles Pandemics: ChatGPT’s Game–Changing Impact on Infectious Disease Control. Ann. Biomed. Eng..

[36] Temsah M., Aljamaan F., Malki K.H., Alhasan K. (2023). ChatGPT and the Future of Digital Health: A Study on Healthcare Workers’ Perceptions and Expectations. Healthcare.

[37] Lukac S., Dayan D., Fink V., Leinert E., Hartkopf A., Veselinovic K., Janni W., Rack B., Pfister K., Heitmeir B. (2023). Evaluating ChatGPT as an Adjunct for the Multidisciplinary Tumor Board Decision–Making in Primary Breast Cancer Cases. Arch. Gynecol. Obstet..

[38] Kavian J.A., Wilkey H.L., Parth A., Boyd C.J. (2023). Harvesting the Power of Arti Fi Cial Intelligence for Surgery: Uses, Implications, and Ethical Considerations. Am. Surg..

[39] Dave T., Athaluri S.A., Singh S. (2023). ChatGPT in Medicine: An Overview of Its Applications, Advantages, Limitations, Future Prospects, and Ethical Considerations. Front. Artif. Intell..

[40] Ruksakulpiwat S., Kumar A., Ajibade A. (2023). Using ChatGPT in Medical Research: Current Status and Future Directions. J. Multidiscip. Healthc..

[41] Tustumi F., Andreollo N.A., Aguilar-Nascimento J.E., No E., Em S., Prazo L. (2023). Future of the Language Models in Healthcare: The Role of Chatgpt. ABCD. Arq. Bras. Cir. Dig..

[42] Kaarre J., Feldt R., Keeling L.E., Dadoo S., Zsidai B., Hughes J.D., Samuelsson K., Musahl V. (2023). Exploring the Potential of ChatGPT as a Supplementary Tool for Providing Orthopaedic Information. Knee Surg. Sports Traumatol. Arthrosc..

[43] Ollivier M., Pareek A., Dahmen J., Kayaalp M.E., Winkler P.W., Hirschmann M.T., Karlsson J. (2023). A Deeper Dive into ChatGPT: History, Use and Future Perspectives for Orthopaedic Research. Knee Surg. Sports Traumatol. Arthrosc..

[44] Sallam M., Salim N.A., Barakat M., Al-Tammemi A.B. (2023). ChatGPT Applications in Medical, Dental, Pharmacy, and Public Health Education: A Descriptive Study Highlighting the Advantages and Limitations. Narra J.

[45] Liberati A., Altman D.G., Tetzlaff J., Mulrow C., Gøtzsche P.C., Ioannidis J.P.A., Clarke M., Devereaux P.J., Kleijnen J., Moher D. (2009). The PRISMA Statement for Reporting Systematic Reviews and Meta-Analyses of Studies That Evaluate Health Care Interventions: Explanation and Elaboration. PLoS Med..

[46] Page M.J., McKenzie J.E., Bossuyt P.M., Boutron I., Hoffmann T.C., Mulrow C.D., Shamseer L., Tetzlaff J.M., Akl E.A., Brennan S.E. (2021). The PRISMA 2020 Statement: An Updated Guideline for Reporting Systematic Reviews. BMJ.

[47] Younis H.A., Ruhaiyem N.I.R., Badr A.A., Abdul-Hassan A.K., Alfadli I.M., Binjumah W.M., Altuwaijri E.A., Nasser M. (2023). Multimodal Age and Gender Estimation for Adaptive Human-Robot Interaction: A Systematic Literature Review. Processes.

[48] Salisu S., Ruhaiyem N.I.R., Eisa T.A.E., Nasser M., Saeed F., Younis H.A. (2023). Motion Capture Technologies for Ergonomics: A Systematic Literature Review. Diagnostics.

[49] Younis H.A., Ruhaiyem N.I.R., Ghaban W., Gazem N.A., Nasser M. (2023). A Systematic Literature Review on the Applications of Robots and Natural Language Processing in Education. Electronics.

[50] Götz S. Supporting Systematic Literature Reviews in Computer Science: The Systematic Literature Review Toolkit. Proceedings of the 21th ACM/IEEE International Conference on Model Driven Engineering Languages and Systems, MODELS 2018.

[51] Muftić F., Kadunić M., Mušinbegović A., Almisreb A.A. (2023). Exploring Medical Breakthroughs: A Systematic Review of ChatGPT Applications in Healthcare. Southeast Eur. J. Soft Comput..

[52] Liao W., Liu Z., Dai H., Xu S., Wu Z., Zhang Y., Huang X., Zhu D., Cai H., Liu T. (2023). Differentiate ChatGPT-Generated and Human-Written Medical Texts. arXiv.

[53] Asch D.A. (2023). An Interview with ChatGPT About Health Care. NEJM Catal..

[54] Li J., Dada A., Kleesiek J., Egger J. (2023). ChatGPT in Healthcare: A Taxonomy and Systematic Review. medRxiv.

[55] Vaishya R., Misra A., Vaish A. (2023). ChatGPT: Is This Version Good for Healthcare and Research?. Diabetes Metab. Syndr. Clin. Res. Rev..

[56] Homolak J. (2023). Opportunities and Risks of ChatGPT in Medicine, Science, and Academic Publishing: A Modern Promethean Dilemma. Croat. Med. J..

[57] Chow J.C.L., Sanders L., Li K. (2023). Impact of ChatGPT on Medical Chatbots as a Disruptive Technology. Front. Artif. Intell..

[58] Aydın Ö., Karaarslan E. (2022). OpenAI ChatGPT Generated Literature Review: Digital Twin in Healthcare. SSRN Electron. J..

[59] Javaid M., Haleem A., Singh R.P. (2023). ChatGPT for Healthcare Services: An Emerging Stage for an Innovative Perspective. BenchCouncil Trans. Benchmarks Stand. Eval..

[60] Liebrenz M., Schleifer R., Buadze A., Bhugra D., Smith A. (2023). Generating Scholarly Content with ChatGPT: Ethical Challenges for Medical Publishing. Lancet Digit. Health.

[61] Eysenbach G. (2023). The Role of ChatGPT, Generative Language Models, and Artificial Intelligence in Medical Education: A Conversation with ChatGPT and a Call for Papers. JMIR Med. Educ..

[62] Alberts I.L., Mercolli L., Pyka T., Prenosil G., Shi K., Rominger A., Afshar-Oromieh A. (2023). Large Language Models (LLM) and ChatGPT: What Will the Impact on Nuclear Medicine Be?. Eur. J. Nucl. Med. Mol. Imag..

[63] Cascella M., Montomoli J., Bellini V., Bignami E. (2023). Evaluating the Feasibility of ChatGPT in Healthcare: An Analysis of Multiple Clinical and Research Scenarios. J. Med. Syst..

[64] Sohail S.S., Farhat F., Himeur Y., Nadeem M., Madsen D.Ø., Singh Y., Atalla S., Mansoor W. (2023). The Future of GPT: A Taxonomy of Existing ChatGPT Research, Current Challenges, and Possible Future Directions. SSRN Electron. J..

[65] Wen J., Wang W. (2023). The Future of ChatGPT in Academic Research and Publishing: A Commentary for Clinical and Translational Medicine. Clin. Transl. Med..

[66] Mohammad B., Supti T., Alzubaidi M., Shah H., Alam T., Shah Z., Househ M. (2023). The Pros and Cons of Using ChatGPT in Medical Education: A Scoping Review. Stud. Health Technol. Inform..

[67] Cox A., Seth I., Xie Y., Hunter-Smith D.J., Rozen W.M. (2023). Utilizing ChatGPT-4 for Providing Medical Information on Blepharoplasties to Patients. Aesthetic Surg. J..

[68] Digiorgio A.M., Ehrenfeld J.M. (2023). Artificial Intelligence in Medicine & ChatGPT: De-Tether the Physician. J. Med. Syst..

[69] Ellaway R.H. (2023). Artificial Scholarship: LLMs in Health Professions Education Research. Adv. Health Sci. Educ..

[70] Samaan J.S., Hui Y., Nithya Y., Lauren R., Stuart H., Wee A., Ng H., Srinivasan N., Park J., Burch M. (2023). Assessing the Accuracy of Responses by the Language Model ChatGPT to Questions Regarding Bariatric Surgery. Obes. Surg..

[71] Gilson A., Safranek C.W., Huang T., Socrates V., Chi L., Street C. (2023). Authors’ Reply to: Variability in Large Language Models’ Responses to Medical Licensing and Certification Examinations. JMIR Med. Educ..

[72] Santandreu-Calonge D., Medina-Aguerrebere P., Hultberg P., Shah M.-A. (2023). Can ChatGPT Improve Communication in Hospitals?. Anu. Thinkepi.

[73] Singh S., Djalilian A., Ali M.J. (2023). ChatGPT and Ophthalmology: Exploring Its Potential with Discharge Summaries and Operative Notes. Semin. Ophthalmol..

[74] Oh N., Choi G., Lee W.Y. (2023). ChatGPT Goes to the Operating Room: Evaluating GPT-4 Performance and Its Potential in Surgical Education and Training in the Era of Large Language Models. Ann. Surg. Treat. Res..

[75] Communication S., Khan R.A., Jawaid M., Khan A.R., Sajjad M. (2023). ChatGPT–Reshaping Medical Education and Clinical Management. Pak. J. Med. Sci..

[76] Liu X., Wu C., Lai R., Lin H., Xu Y., Lin Y., Zhang W. (2023). ChatGPT: When the Artificial Intelligence Meets Standardized Patients in Clinical Training. J. Transl. Med..

[77] Gao C.A., Howard F.M., Pearson A.T., Dyer E.C. (2023). Comparing Scientific Abstracts Generated by ChatGPT to Real Abstracts with Detectors and Blinded Human Reviewers. NPJ Digit. Med..

[78] Rahimzadeh V., Kostick-quenet K., Barby J.B., Mcguire A.L., Rahimzadeh V., Kostick-quenet K., Barby J.B., Rahimzadeh V., Kostick-Quenet K., Barby J.B. (2023). Ethics Education for Healthcare Professionals in the Era of ChatGPT and Other Large Language Models: Do We Still Need It? Ethics Education for Healthcare Professionals in the Era of ChatGPT And. Am. J. Bioeth..

[79] Lahat A., Shachar E., Avidan B., Glicksberg B., Klang E. (2023). Evaluating the Utility of a Large Language Model in Answering Common Patients’ Gastrointestinal Health-Related Questions: Are We There Yet?. Diagnostics.

[80] Wang H., Jenny Y., Yuan D. (2023). Future of ChatGPT in Pharmacovigilance. Drug Saf..

[81] Rodigin A. (2023). Is Medicine Ready for ChatGPT–Why Not Just Ask ChatGPT?. Eur. J. Transl. Clin. Med..

[82] Wang H., Wu W., Dou Z., He L., Yang L. (2023). Performance and Exploration of ChatGPT in Medical Examination, Records and Education in Chinese: Pave the Way for Medical AI. Int. J. Med. Inform..

[83] Cheng K., Li Z., He Y., Guo Q., Lu Y., Gu S., Wu H. (2023). Potential Use of Artificial Intelligence in Infectious Disease: Take ChatGPT as an Example. Ann. Biomed. Eng..

[84] Nov O., Singh N., Mann D. (2023). Putting ChatGPT’s Medical Advice to the (Turing) Test: Survey Study. JMIR Med. Educ..

[85] Janamala V., Sai I., Suresh R., Daram B. (2023). Realization of Green 5G Cellular Network Role in Medical Applications: Use of ChatGPT–AI. Ann. Biomed. Eng..

[86] Janamla V., Babu S., Patil D., Nagaraja R.C.H. (2023). Response of ChatGPT for Humanoid Robots Role in Improving Healthcare and Patient Outcomes. Ann. Biomed. Eng..

[87] Polonsky M.J., Rotman J.D. (2023). Should Artificial Intelligent Agents Be Your Co-Author? Arguments in Favour, Informed by ChatGPT. Australas. Mark. J..

[88] Sedaghat S. (2023). Success Through Simplicity: What Other Artificial Intelligence Applications in Medicine Should Learn from History and ChatGPT. Ann. Biomed. Eng..

[89] Bin Arif T., Munaf U., Ul-Haque I. (2023). The Future of Medical Education and Research: Is ChatGPT a Blessing or Blight in Disguise?. Med. Educ. Online.

[90] Wornow M., Xu Y., Thapa R., Patel B., Steinberg E., Fleming S. (2023). The Shaky Foundations of Large Language Models and Foundation Models for Electronic Health Records. NPJ Digit. Med..

[91] Hügle T. (2023). The Wide Range of Opportunities for Large Language Models Such as ChatGPT in Rheumatology. RMD Open.

[92] Liu J., Wang C., Liu S. (2023). Utility of ChatGPT in Clinical Practice. J. Med. Internet Res..

[93] Cohen I.G., Cohen I.G. (2023). What Should ChatGPT Mean for Bioethics? What Should ChatGPT Mean for Bioethics?. Am. J. Bioeth..

