# Supplementary material for: A Systematic Review and Meta-Analysis of Artificial Intelligence Tools in Medicine and Healthcare: Applications, Considerations, Limitations, Motivation and Challenges
Source: Diagnostics (Basel). 2024 Jan 4;14(1):109. doi: 10.3390/diagnostics14010109 (PMC10802884; doi:10.3390/diagnostics14010109)
Supplement: Supplementary file 1 [file diagnostics-14-00109-s001.zip › diagnostics-2716439-PRISMA(FINAL).pdf]

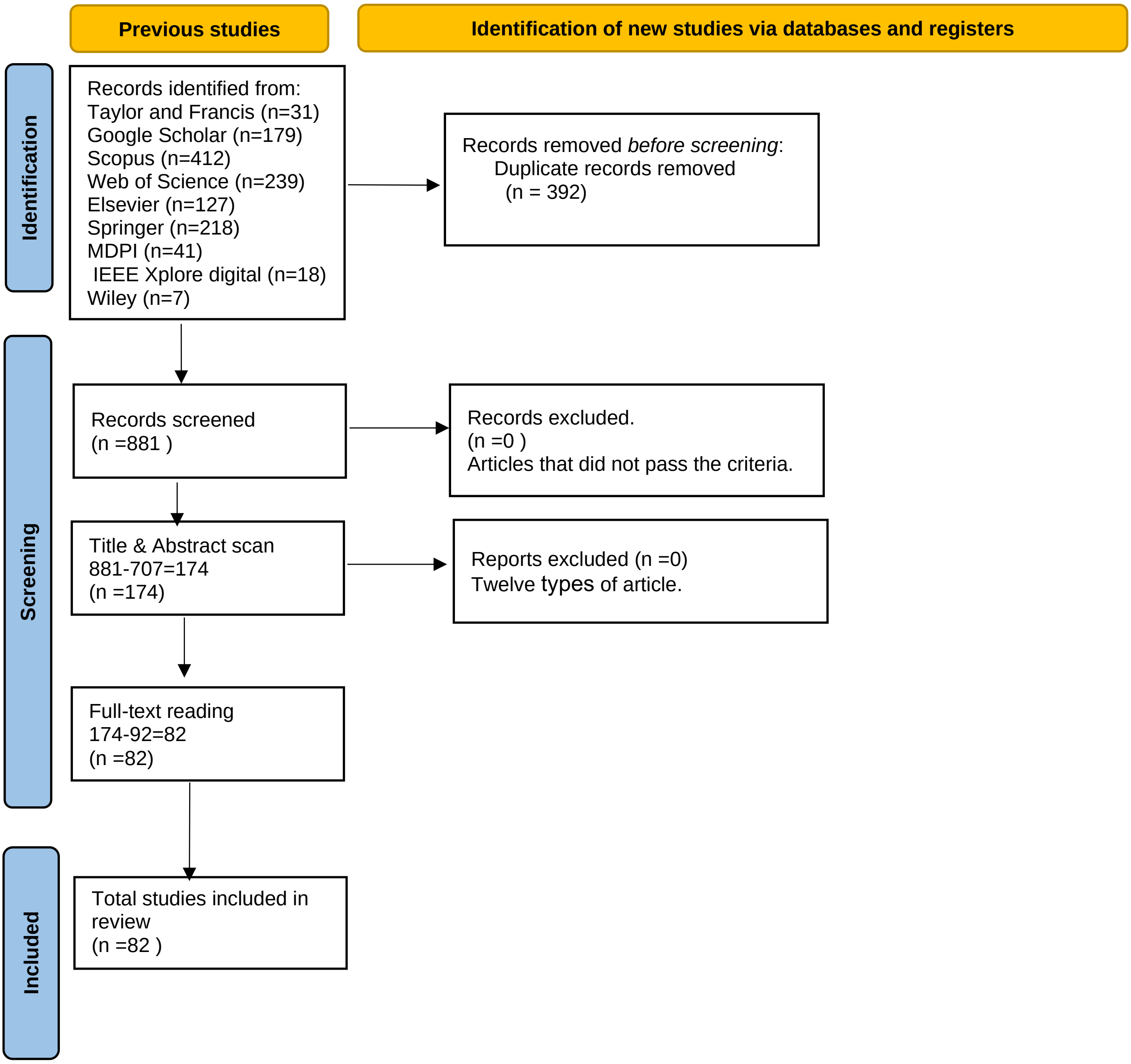

Figure S1. PRISMA 2020 flow diagram for updated systematic reviews which included searches of databases and registers only

*Consider, if feasible to do so, reporting the number of records identified from each database or register searched (rather than the total number across all databases/registers).

**If automation tools were used, indicate how many records were excluded by a human and how many were excluded by automation tools.

From: Page MJ, McKenzie JE, Bossuyt PM, Boutron I, Hoffmann TC, Mulrow CD, et al. The PRISMA 2020 statement: an updated guideline for reporting systematic reviews. BMJ 2021;372:n71. doi: 10.1136/bmj.n71

For more information, visit: http://www.prisma-statement.org/
